# A Case of Metachronous Prostate Adenocarcinoma and Chromophobe Renal Cell Carcinoma in a Nigerian Male Patient: Diagnostic and Therapeutic Challenges

**DOI:** 10.7759/cureus.100211

**Published:** 2025-12-27

**Authors:** Olurotimi J Badero, Christiana Ogunlana, Adewumi Alabi, Victor Isibor

**Affiliations:** 1 Department of Interventional Cardiology, Iwosan Lagoon Hospital, Lagos, NGA; 2 Department of Radiotherapy, Lagos University Teaching Hospital, Lagos, NGA

**Keywords:** case report, chromophobe renal cell carcinoma, hdr brachytherapy, immunohistochemistry, multimodal therapy, prostate adenocarcinoma

## Abstract

Prostate adenocarcinoma and chromophobe renal cell carcinoma (ChRCC) are two distinct urologic malignancies with different prognoses and treatment approaches. The occurrence of both tumors in the same individual is highly unusual. We describe the case of a 69-year-old man with a long-standing history of hypertension who had previously undergone prostatectomy for benign prostatic hyperplasia. In 2022, he re-presented with recurrent lower urinary tract symptoms. Biopsy confirmed acinar adenocarcinoma of the prostate (Gleason 4+3), and magnetic resonance imaging (MRI) staged the disease as T3aN0M0. He was treated with high-dose-rate (HDR) brachytherapy followed by external beam radiotherapy (EBRT). Two years later, a right renal mass was detected. While the initial histology favored oncocytoma, immunohistochemistry revealed strong cytokeratin (CK)7 expression with focal CD117 staining, confirming the eosinophilic subtype of ChRCC. This case illustrates the importance of thorough histopathologic work-up of renal lesions, the diagnostic value of immunohistochemistry, and the need for careful follow-up in patients with complex urologic histories.

## Introduction

Prostate adenocarcinoma is the most frequently diagnosed non-cutaneous malignancy in men. By contrast, chromophobe renal cell carcinoma (ChRCC) accounts for only 5-7% of renal cell carcinomas [1]. Their coexistence in the same patient is rare and creates additional diagnostic and therapeutic challenges. We present a case of sequential prostate adenocarcinoma and chromophobe RCC in a Nigerian male patient, emphasizing the role of immunohistochemistry and the relevance of multimodal treatment.

## Case presentation

A 69-year-old man with hypertension diagnosed three decades earlier was referred for external beam radiation therapy (EBRT) after already receiving high-dose rate (HDR) brachytherapy for prostate cancer. He denied smoking, alcohol use, or a family history of cancer. His past medical record was negative for diabetes, asthma, seizure disorders, or sickle cell disease. He had remained generally well until 10 years earlier, when he developed bladder outlet obstruction (BOO). A simple prostatectomy performed at that time reportedly demonstrated benign histology, with no evidence of malignancy on histopathological examination.

**Figure 1:**
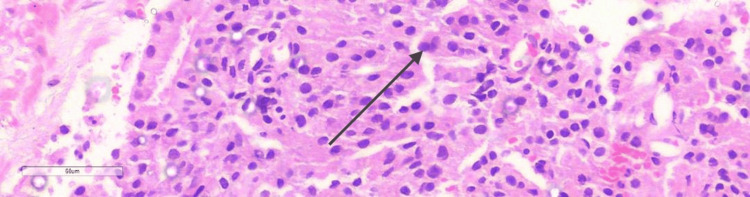
Photomicrograph of prostatic tissue showing tumour composed of malignant glands of varying shapes and sizes forming cribriform patterns. The glands are lined by single layer of mildly atypical cells with enlarged hyperchromatic nuclei (black arrow). Magnification: 4x 400; Stain used: hematoxylin and eosin

Seven years later, in 2022, he again developed BOO symptoms. A prostrate biopsy revealed acinar adenocarcinoma with a Gleason score of 4+3 (International Society of Urological Pathology (ISUP) grade group 3), as shown in Figure 1.

**Figure 2:**
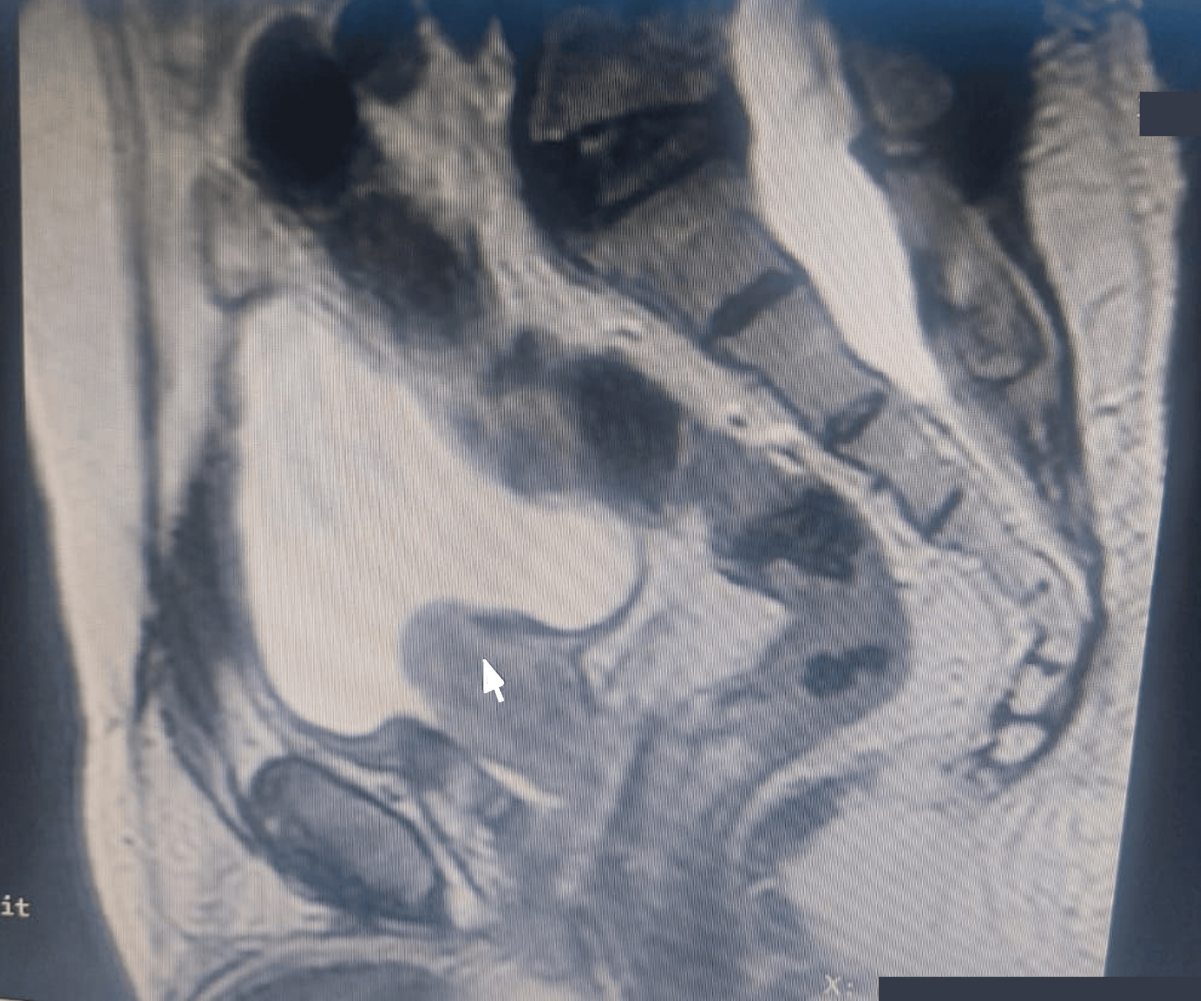
Pelvic MRI showing enlarged prostate (white arrow)

Magnetic resonance imaging (MRI) of the pelvis revealed extra-prostatic extension without nodal disease (T3aN0M0) as shown in Figure 2. Computed tomography (CT) scan of the abdomen and pelvis excluded liver metastases, and a bone scan ruled out skeletal involvement.

Two months prior to presentation, he received HDR brachytherapy to the prostatic bed, 27 Gy in two fractions (13.5 Gy each). At presentation, he was then scheduled for EBRT to the prostate, 50 Gy in 25 fractions.

**Figure 3:**
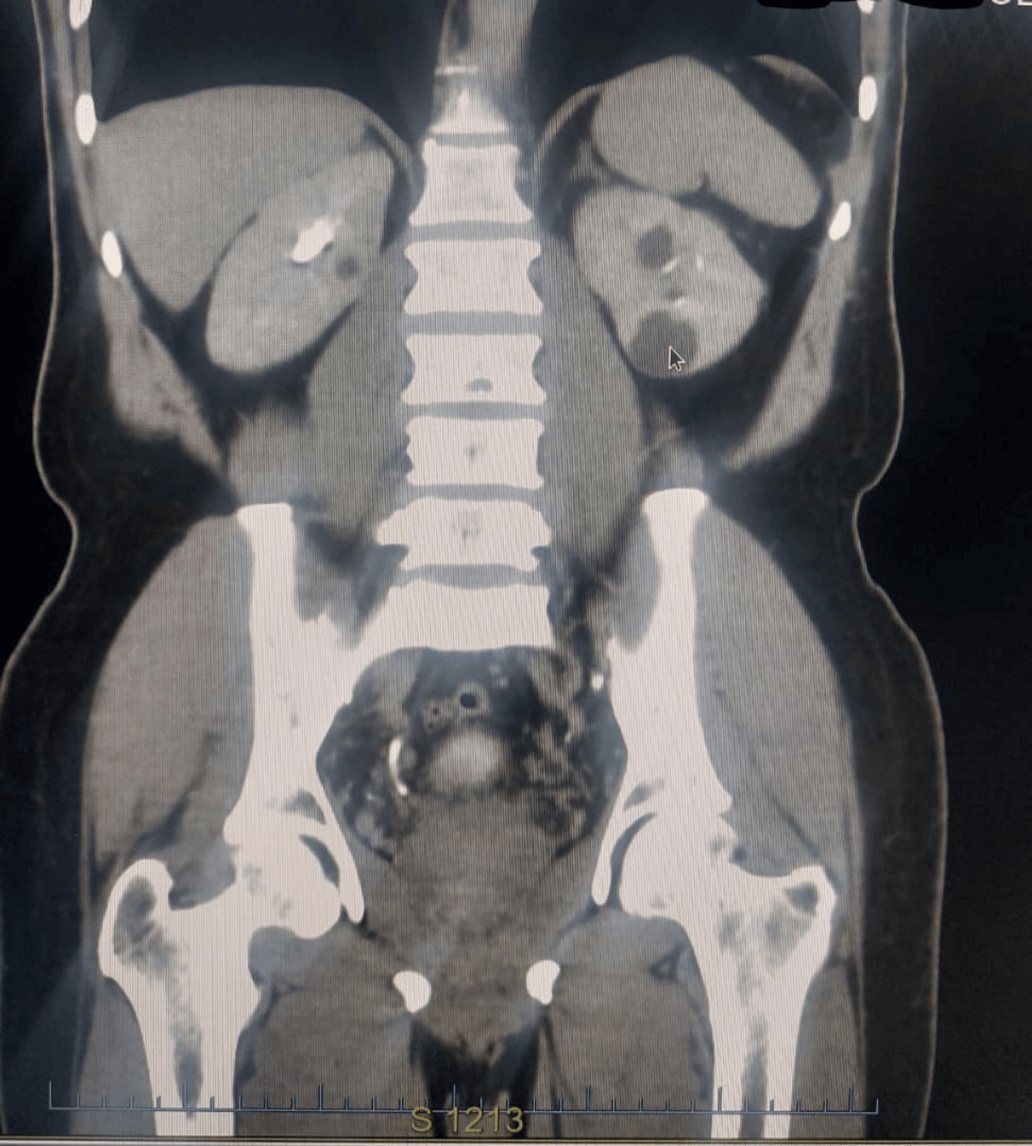
Abdominopelvic CT scan prior to nephrectomy

Approximately one year before the current presentation for prostate cancer radiotherapy, the patient underwent a right nephrectomy for a renal mass. Abdominopelvic CT scan done at the time showed a renal mass (Figure 3). The initial histopathology suggested oncocytoma, but immunohistochemistry revealed cytokeratin (CK)7 positivity with focal CD117 expression, leading to a final diagnosis of eosinophilic variant ChRCC. There were no adverse histologic features such as sarcomatoid change, microscopic necrosis, or vascular invasion. Table 1 shows a list of the investigations carried out.

**Table 1:** Investigations ISUP: International Society of Urological Pathology

Date	Investigation	Result
Three years prior to presentation	Prostate biopsy	Adenocarcinoma, Gleason 4+3=7, ISUP Grade 3
At presentation	Pelvic MRI	Extra-prostatic extension
At presentation	Abdominopelvic CT	No liver or bone metastases
At presentation	Bone scan	No osteoblastic metastases
At presentation	Renal histology and Immunohistochemistry	CK7: Strongly positive CD117: Rare foci

A diagnosis of prostate adenocarcinoma coexisting with chromophobe RCC was made. The treatments administered for the respective malignancies are listed in Table 2.

**Table 2:** Treatment

Date	Malignancy	Treatment given
A year prior to presentation	Renal cancer	Right nephrectomy
Two months prior to presentation	Prostate cancer	High Dose Rate Brachytherapy: 27 Gray in 2 fractions
Ongoing at the time of the report	Prostate cancer	External Beam Radiation Therapy: 50 Gray in 25 fractions

At the time of reporting, the patient was clinically stable, still undergoing EBRT, and showed no evidence of metastatic spread on imaging. He remains under active follow-up.

## Discussion

The development of synchronous or metachronous tumors within the genitourinary tract is unusual, and such cases can present diagnostic dilemmas. Our patient first developed prostate adenocarcinoma, followed later by ChRCC; two biologically distinct tumors requiring different management strategies.

Prostate cancer is the most common solid tumor in men, and prognosis is closely linked to Gleason grading. Gleason 7 cancers, especially 4+3 patterns, carry a worse prognosis than 3+4 disease, necessitating more aggressive treatment [2]. In this patient, extra-prostatic extension warranted multimodal therapy, and he was managed with HDR brachytherapy followed by EBRT. HDR brachytherapy has demonstrated excellent local control and survival benefits both as primary and salvage therapy [3,4]. Combined modality approaches have further improved outcomes in high-risk disease [5].

ChRCC, though uncommon, generally has a more favorable prognosis than clear cell RCC provided that high-risk features such as sarcomatoid differentiation or necrosis are absent [6,7]. Accurate diagnosis is critical because management and prognosis differ significantly from those of other renal tumors. The eosinophilic variant of ChRCC can resemble renal oncocytoma on routine hematoxylin and eosin (H&E) staining, making diagnosis difficult. Immunohistochemistry, particularly CK7 positivity and variable CD117 expression, is extremely helpful in distinguishing the two [7,8]. This distinction has major clinical implications: oncocytoma is benign, whereas ChRCC requires surgical excision and long-term surveillance.

The ISUP grading system has refined prognostic classification for certain subtypes of RCC, while the World Health Organization (WHO) classification further integrates molecular and immunohistochemical features [9,10].

This case highlights several learning points. Recurrent urinary obstruction in a patient with prior prostate surgery should prompt reevaluation with biopsy, as earlier histologic testing may have missed malignancy. Second, multimodal therapy offers effective disease control in high-risk prostate cancer. Finally, the accurate diagnosis of renal tumors relies on following up histology with immunohistochemistry to avoid misclassification and inappropriate management [10].

## Conclusions

This case highlights the importance of maintaining vigilance in patients with previous urologic interventions who develop new or recurrent symptoms. Beyond histology, the variation between oncocytoma and ChRCC requires immunohistochemical confirmation, as management differs significantly. For prostate cancer with extra-prostatic spread, timely multimodal therapy remains crucial for disease control.
